# Application of microsatellite loci on the chromosome X for rapid prenatal detection of the chromosome X numerical abnormalities

**DOI:** 10.3325/cmj.2011.52.392

**Published:** 2011-06

**Authors:** Kristina Crkvenac Gornik, Zorana Grubić, Katarina Štingl, Ivana Tonković Đurišević, Davor Begović

**Affiliations:** 1 Division of Genetics, Department of Pediatrics University Hospital Center, Zagreb, Croatia; 2 Tissue Typing Center, University Hospital Center, Zagreb, Croatia

## Abstract

**Aim:**

To determine the value of short-tandem repeat markers on the chromosome X (X-STR) for prenatal diagnostics of the chromosome X numerical disorders.

**Methods:**

We investigated the genetic variability of 5 X-markers (DXS9895, DXS6810, DXS6803, GATA172D05, and HPRTB) in 183 healthy Croatian individuals (90 men and 93 women). We also tested 13 patients with X chromosome disorders (Turner syndrome – 6 cases; Klinefelter syndrome – 5 cases, and Triple X syndrome – 2 cases). The analysis was performed using polymerase chain reaction amplification with specific primers and electrophoresis on a polyacrylamide gel. The study was performed in 2010.

**Results:**

Our sample showed no significant differences in allelic frequencies of the investigated X-markers from other European populations. A set of 5 X-STR markers was sufficiently informative for a successful determination of the chromosome X numerical abnormalities.

**Conclusion:**

Since no false positive or negative results were observed, diagnostic value of the investigated X-STR loci for prenatal detection of chromosome X numerical disorders was confirmed. Our study represents an important step toward an improved prenatal diagnostics in Croatia.

The analysis of short tandem repeat (STR) markers using polymerase chain reaction (PCR) method has become a widely applied technique in forensic individual identification, rapid detection of chromosome aneuploidies in prenatal and postnatal diagnosis, as well as paternity testing (1-5). Until now, a large number of autosomal and Y-chromosomal markers has been forensically evaluated and used for various purposes. Although X-chromosomal markers have been increasingly applied in both forensic and medical field, their role has not been as extensively investigated as that of autosomal and Y-chromosomal markers. Several investigations have documented the accuracy of fluorescent PCR using STR loci for the rapid prenatal diagnoses of numerical disorders affecting the chromosomes 21, 18, and 13 (6-8). However, the low polymorphism of the most chromosome X and Y markers has hampered the use of the PCR-STR approach for the detection of numerical disorders of sex chromosomes, such as the Turner (45, X) or Klinefelter (47, XXY) syndromes (1,7). A few years ago, a group of authors reported on the application of PCR-STR method in the detection of X-chromosome abnormalities (9,10). One of the biggest challenges is the Turner syndrome, in which a sufficient number of STR loci has to be included to be sure that the individual has only a single X chromosome. The aims of the present study were to investigate the diagnostic informativeness of 5 X-linked STR markers: DXS9895 (Xpter-Xp22.1), GATA172D05 (Xq26.1), DXS6810 (Xq12-Xq21.33), DXS6803 (Xq24-Xq27), and HPRTB (Xq27.3) in the Croatian population and to evaluate the diagnostic value of these 5 loci.

## Participants and methods

We used blood samples from 183 unrelated healthy individuals (90 men and 93 women) from the Croatian population (citizens of Zagreb), available in the Tissue Typing Center of the University Hospital Center. All participants gave a written informed consent and filled out a questionnaire on their demographic characteristics and medical background. Their anonymity was ensured by giving each participant a code number. Hundred and fifty samples had been previously tested by conventional cytogenetic analysis. In this group, there were also 13 samples with X chromosome disorders (2 with 47, XXX; 6 with 45, X; and 5 with 47, XXY). The analysis of all participants was performed by PCR-STR method. Genomic DNA was isolated from peripheral blood by NucleoSpin Blood isolation kit (Machery-Nagel, Duran, Germany) (11). The study was performed in 2010. Ethical approval was received from the Ethics Committee of the University of Zagreb School of Medicine.

Amplification was performed in a total volume of 11.5 µL in Mastercycler gradient thermocyler (Eppendorf, Duran, Germany) as previously described (12,13). After amplification, the PCR products (1.5 µL) were mixed with 3 µL of loading buffer and 1 µL of each of 2 internal size markers and applied on gel. On each gel, we also included one external, commercial size marker. Electrophoresis was performed using a 6% standard denaturing polyacrylamide gel in an automated laser fluorescence DNA sequencer (ALFexpress, Pharmacia Biotech, Uppsala, Sweden). The amplification products were analyzed and their relative fluorescent intensities calculated using AlleleLocator software (Pharmacia Biotech). The assignment of alleles was performed using allelic ladders.

The Hardy-Weinberg equilibrium test was performed by χ^2^ test. Allele and genotype frequencies for each X-STR locus were determined by direct counting. The power of exclusion was calculated as described by Crow (14), while polymorphism information content (PIC) value was obtained as suggested by Hearne (15). The average power of discrimination (PD) was estimated as proposed by Desmarais (16).

## Results and discussion

Allele frequencies of 5 X-linked microsatellites in the Croatian population were calculated separately for men and women, and for all participants together (Table 1), and no sex-related differences were found. DXS6803 locus was most polymorphic with 15 different alleles, while DXS6810 locus was least polymorphic with only 6 different alleles. The population study (n = 183) confirmed that all 5 STR loci were informative, which is in concordance with the data from other populations (17-19).

**Table 1:** Distribution of allele frequencies at DXS6810, HPRTB, DXS9895, DXS6803, and GATA172D05 loci in the Croatian population (n = 183)

DXS6810		HPRTB		DXS9895		DXS6803		GATA172D05	
alleles	frequency	alleles	frequency	alleles	frequency	alleles	frequency	alleles	frequency
1	-	6	-	11	0.0254	9	0.0072	6	0.1413
2	0.0072	7	-	12	0.0797	10	0.0326	7	0.0290
3	0.0543	8	0.0072	13	0.2065	11	0.1993	8	0.1232
4	0.3261	9	0.0435	14	0.2174	11.3	0.0652	9	0.0870
5	0.4130	10	0.0543	15	0.2029	12	0.1884	10	0.2030
6	0.1812	11	0.1159	16	0.2174	12.3	0.1522	11	0.2500
7	0.0181	12	0.239	17	0.0435	13	0.1014	12	0.1449
		13	0.2790	18	0.0072	13.3	0.1703	13	0.0217
		14	0.1413			14	0.0036		
		15	0.1087			14.3	0.0580		
		16	0.0109			15.3	0.0181		
		17	-			16.3	0.0036		

The observed statistical parameters of the examined X-linked markers are shown in Table 2. PIC was calculated from the combined data for men and women, whereas observed heterozygosity (Hobs), genotypes, matching probability, and power of exclusion were obtained only from data for women. No deviation from Hardy-Weinberg equilibrium was found. Four loci (DXS9895, GATA172D05, DXS6803, and HPRTB) showed high PIC value and high Hobs value. The PIC value of the examined markers ranged from 0.623 (DXS6810) to 0.901 (DXS6803). The DXS6810 showed a Hobs value lower than 0.60, which corresponds to the high frequency of the allele 5 in our sample.

**Table 2:** Statistical parameters for short tandem repeat markers HPRTB, DXS6810, DXS6803, GATA172D05, and DXS9895 obtained from the population study (N = 183).

	HPRTB	DXS6810	DXS6803	GATA172D05	DXS9895
Observed heterozygosity in women	0.656	0.591	0.849	0.731	0.656
Expected heterozygosity in men	0.797	0.609	0.895	0.850	0.781
Polymorphism information content	0.805	0.623	0.901	0.857	0.795
Power of discrimination in men	0.799	0.708	0.894	0.871	0.816
Average power of discrimination in women	0.925	0.740	0.979	0.958	0.918
Expected probability of exclusion in women ¶	0.944	0.836	0.982	0.967	0.935
Matching probability	0.056	0.164	0.017	0.033	0.065

Among the successfully processed 150 samples, sex chromosomal aneuploidies were diagnosed by standard cytogenetic methods in 13 cases (Table 3). For each sample with X chromosome disorders, we also performed a PCR-STR analysis using these 5 STR loci. All 6 samples from patients with Turner syndrome had only one fluorescent peak (Table 2). At the same time, all 5 patients with karyogram 47, XXY had 2 alleles at DXS9895 and DXS6803, and 2 or 1 allele at the other 3 STR loci. It is important to mention that for all samples from this subgroup at least 2 tested X-microsatellites showed 2 different alleles. Both samples of Triple X syndrome demonstrated 3 alleles at DXS9895 and a double dose of 1 or 2 alleles at the remaining 4 loci (two times 1 or 2 alleles at the other 4 STR).

**Table 3:** Thirteen samples from patients with X chromosome disorders tested by 5 X-short tandem repeat markers

Sample	HPRTB	DXS6810	DXS6803	GATA172D05	DXS9895
**Kariotype 45, X**					
1.	12	5	12	12	13
2.	14	4	13.3	11	16
3.	12	5	11	11	16
4.	11	4	12.3	10	16
5.	13	5	12	11	14
6.	13	5	11	10	15
**Kariotype 47, XXY**					
7.	13	5	10, 12	10	13, 16
8.	12, 13	6	11, 12	8, 11	14, 17
9.	12	4	12.3, 13.3	10, 12	14, 16
10.	12, 13	3,6	11, 12	10, 11	15, 16
11.	12	4,5	11, 13.3	11	13, 14
**Kariotype 47,XXX**					
12.	12*, 13	4*, 5	11, 12*	10*, 12	15, 16, 17
13.	14	4, 5*	11.3, 12, 13	10, 11*	14, 15, 18

*Trisomic diallelic.

Our results are in agreement with the results of other studies demonstrating the usefulness of PCR-STR method for detection of X chromosomal disorders (9,10,20). The method is fast, sensitive, and suitable for screening of a large number of samples. In conclusion, data on X-chromosome STRs among Croatians would be useful for paternity testing and forensic purposes but also for X-chromosome mapping studies. The set of 5 STR loci tested in the present study showed to be sufficient for the diagnosis of Turner, Klinefelter, and Triple X syndrome.
